# P88 Enhancing host-microbiota synergy: fruit by-product polyphenols as prebiotic adjuvants to prevent pathogenic *Escherichia coli* colonization

**DOI:** 10.1093/jacamr/dlag102.094

**Published:** 2026-06-26

**Authors:** Bryan Moreno-Chamba, Julio Salazar-Bermeo, María Concepción Martínez-Madrid, Domingo Saura, Manuel Valero, Victoria Lizama, Nuria Martí

**Affiliations:** Instituto Universitario de Ingeniería de Alimentos-FoodUPV, Universitat Politècnica de València, 46022, Valencia, Spain; Instituto de Investigación, Desarrollo e Innovación en Biotecnología Sanitaria de Elche (IDiBE), Edificio Torregaitán, Universidad Miguel Hernández de Elche (UMH), 03202, Alicante, Spain; Instituto Universitario de Ingeniería de Alimentos-FoodUPV, Universitat Politècnica de València, 46022, Valencia, Spain; Instituto de Investigación, Desarrollo e Innovación en Biotecnología Sanitaria de Elche (IDiBE), Edificio Torregaitán, Universidad Miguel Hernández de Elche (UMH), 03202, Alicante, Spain; Instituto de Investigación, Desarrollo e Innovación en Biotecnología Sanitaria de Elche (IDiBE), Edificio Torregaitán, Universidad Miguel Hernández de Elche (UMH), 03202, Alicante, Spain; Instituto de Investigación, Desarrollo e Innovación en Biotecnología Sanitaria de Elche (IDiBE), Edificio Torregaitán, Universidad Miguel Hernández de Elche (UMH), 03202, Alicante, Spain; Instituto de Investigación, Desarrollo e Innovación en Biotecnología Sanitaria de Elche (IDiBE), Edificio Torregaitán, Universidad Miguel Hernández de Elche (UMH), 03202, Alicante, Spain; Instituto Universitario de Ingeniería de Alimentos-FoodUPV, Universitat Politècnica de València, 46022, Valencia, Spain; Instituto de Investigación, Desarrollo e Innovación en Biotecnología Sanitaria de Elche (IDiBE), Edificio Torregaitán, Universidad Miguel Hernández de Elche (UMH), 03202, Alicante, Spain

## Abstract

**Background:**

Antimicrobial resistance (AMR) is an increasing global health concern, driven in part by the reduced effectiveness of antibiotics against gut pathogens. *Escherichia coli* remains a common cause of gastrointestinal infections, and its treatment often involves antibiotics that can also disrupt beneficial gut microbiota. This highlights the need for alternative strategies that do not rely on killing bacteria directly, an approach that can promote resistance, but instead support beneficial microbes in outcompeting pathogens. Fruit by-products, which are rich in anthocyanins and other phenolic compounds, offer a sustainable source of bioactive molecules. These compounds may help shape the gut environment by supporting protective microbial communities that limit pathogen attachment to the intestinal lining.

**Objectives:**

This study explored the potential of phenolic extracts from grape (GRE), cherry (CHE), and strawberry (SWE) by-products to act as prebiotic agents. Specifically, we assessed whether these extracts could promote beneficial bacteria while reducing the ability of pathogenic *E. coli* to colonize.

**Methods:**

Non-inhibitory concentrations of extracts were determined by a microdilution assay and were used for the experiments. The effect of the extracts was evaluated on fitness of *Bifidobacterium longum* by measuring biofilm formation by crystal violet staining. A Prebiotic Activity Score (PAS) was also calculated to assess selectivity in favour of beneficial *B. bifidum*, over *E. coli,* through a growth curve assay. The short-chain fatty acids production by extract-treated *B. longum* were determined by HPLC-DAD. Finally, a competitive adhesion assay using Caco-2 intestinal epithelial cells was performed to model how these extracts influence bacterial attachment in a host-like environment.

**Results:**

After confirming the inhibitory concentration of extracts against *B. longum* and *E. coli*, the extracts managed to promote the initial biofilm formation of *B. longum*. SWE promoted the preformed biofilm of *B. longum* while CHE improved mature biofilm of *B. longum* (*P<*0.05). After a growth curve assay, all samples showed positive PAS but with no statistical differences (*P>*0.05), displaying a selective effect promoting the growth of *B. longum* instead of *E. coli*. This was translated in a high lactate, acetate and butyrate production by *B. longum* after incubation (*P<*0.05). Ultimately, the PAS results were reflected in a competitiveness assay, where the samples showed a noted effect reducing the adhesion of *E. coli*, especially during competition with *B. longum* (*P<*0.05), improving the effect of the beneficial strain.

**Conclusions:**

Anthocyanin-rich extracts from grape, cherry, and strawberry by-products demonstrate significant potential as functional ingredients for gut health maintenance. These extracts promote the growth, adhesion, and biofilm formation of *Bifidobacterium longum*, while maintaining selective activity against pathogenic *Escherichia coli*, as indicated by positive PAS. In competitive adhesion assays utilizing Caco-2 intestinal epithelial cell models, the presence of these extracts enhances the competitive ability of *B. longum*, effectively reducing *E. coli* attachment. These data confirm the capacity of the extracts to modulate both microbiota composition and host–microbe interactions, supporting their utility as nutraceutical agents to strengthen the intestinal barrier and limit pathogenic colonization.

